# Machine vision benefits from human contextual expectations

**DOI:** 10.1038/s41598-018-38427-0

**Published:** 2019-02-14

**Authors:** Harish Katti, Marius V. Peelen, S. P. Arun

**Affiliations:** 1 Centre for Neuroscience, Indian Institute of Science, Bangalore, 560012 India; 2 Donders Institute for Brain, Cognition and Behaviour in Nijmegen, Nijmegen, The Netherlands

## Abstract

Scene context is known to facilitate object recognition in both machines and humans, suggesting that the underlying representations may be similar. Alternatively, they may be qualitatively different since the training experience of machines and humans are strikingly different. Machines are typically trained on images containing objects and their context, whereas humans frequently experience scenes without objects (such as highways without cars). If these context representations are indeed different, machine vision algorithms will be improved on augmenting them with human context representations, provided these expectations can be measured and are systematic. Here, we developed a paradigm to measure human contextual expectations. We asked human subjects to indicate the scale, location and likelihood at which cars or people might occur in scenes without these objects. This yielded highly systematic expectations that we could then accurately predict using scene features. This allowed us to predict human expectations on novel scenes without requiring explicit measurements. Next we augmented decisions made by deep neural networks with these predicted human expectations and obtained substantial gains in accuracy for detecting cars and people (1–3%) as well as on detecting associated objects (3–20%). In contrast, augmenting deep network decisions with other conventional computer vision features yielded far smaller gains. Taken together, our results show that augmenting deep neural networks with human-derived contextual expectations improves their performance, suggesting that contextual representations are qualitatively different in humans and deep neural networks.

## Introduction

*We work with being, but non-being is what we use*.

- *Tao Te Ching*^1^

Detecting targets in real world scenes remains a hard problem even for the hugely successful deep convolutional neural networks (CNNs). For instance, state-of-the-art deep convolutional networks such as the Alexnet^2^ and Faster RCNN^3^ can detect people with 82–88% accuracy and cars with 77–84% top-1 accuracy based on our evaluation on a real world scene dataset^4^. In contrast, humans fare much better at 93% in speeded car detection or person detection tasks^5^. One potential reason for this performance gap is that humans and machines have qualitatively different training data. Machines are typically trained on large image databases containing targets embedded in their surrounding context. When the object has a weak correlation with its surrounding context, the context signal can be difficult to learn in the presence of the vastly more informative object features. When the object has a strong correlation with its context (such as kitchen scenes that always contain microwave ovens), the object signal can potentially be learned erroneously along with the context. These variations make it difficult to learn context systematically and independently of object features. In contrast, humans often view scenes in which the target object moves out of view or moves against a static background. Such experiences are an opportunity for humans to learn separate features for target and context. If this is true, it follows that humans must have systematic expectations about target objects even on scenes that do not contain those targets. These differences in visual experience could potentially lead to qualitatively different contextual representations in machines and humans. If this were true, it follows that their performance can be improved by augmenting them with human-derived contextual expectations.

That context can aid in object detection has been appreciated both in studies of human vision as well as computer vision. In humans, it is well known that finding objects in a congruent context is faster than in an incongruent context^6,7^. Brief previews of scenes guide eye movements towards cued targets^8^. Both nontarget objects and coarse scene layout contribute to object detection^9–12^ although their relative contributions have only been elucidated recently^5^. In the brain, there are dedicated scene processing regions^13^ that respond to scenes as well as to their associated objects^14,15^. In computer vision, contextual priors learnt from target present scenes have been used to improve object detection and localisation by constraining the locations to search^16–18^. Models incorporating contextual features have also been shown to be useful in predicting task directed eye-movements^19^. More recently, deep convolutional networks have shown dramatic improvements in scene^20^ and object classification^21^. However, it is not clear whether these deep networks learn target and/or context features. Thus, while there is evidence that scene context can facilitate object detection in both machines and humans, it is largely thought to facilitate searching for objects. Furthermore, whether context involves processing target features, associated nontarget objects, and/or scene layout has remained unclear.

## Results

Our central premise was that machines and humans have qualitatively different context representations. We selected cars and people as suitable test categories because they are ecologically important, extensively researched^22–24^ and common in popular datasets^20,25–27^. Our results are organized as follows: We first performed a behavioural experiment on humans in which we measured their contextual expectations on natural scenes and used computational modelling to understand and predict these expectations. Second, we demonstrate that these predicted human expectations can be used to improve the performance of state-of-the-art object detectors. Finally, we demonstrate that this improvement is non-trivial in that it cannot be obtained using target-related signals of various types. To facilitate further research, the code, behavioural data, visual features and stimuli used for this study are publicly available at https://github.com/harish2006/cntxt_likelihood.

### Measuring human expectations (Experiment 1)

If humans can process object features independently of context, then they must be able to form systematic expectations about the likelihood, scale and location of where objects might occur in a scene. Here we set out to measure these expectations systematically using a behavioural experiment on human subjects. On each trial, subjects were shown a scene that did not contain cars or people, and were asked to indicate the likelihood, scale and location of cars or people in the scene at a later point in time (see Methods for details).

**Figure 1:**
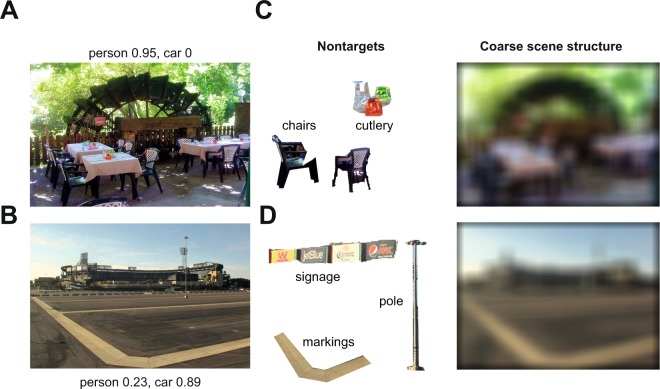
(**A**) Example scene rated by subjects as likely to contain people but not cars. (**B**) Example scene with high car and low person likelihood (**C**,**D**) show representative nontarget objects and coarse scene information extracted from these images. We modeled these expectations using person/car features (not shown), nontarget objects (*middle*) and coarse scene structure (*right)*. Image sources (**A**) Wikimedia (https://commons.wikimedia.org/wiki/File:Fontaine-de-Vaucluse_Terrasse_du_restaurant_P%C3%A9trarque_et_Laure.JPG, Marianne Casamance, CC BY-SA 4.0, https://creativecommons.org/licenses/by-sa/4.0/, no changes made) and (**B**) Wikimedia (https://commons.wikimedia.org/wiki/File:Angel_Stadium_with_parking_lot.jpg, Downtowngal, CC BY-SA 3.0, https://creativecommons.org/licenses/by-sa/3.0/, no changes made), please click on the hyperlinks to open original images in a browser. Coarse scene information and nontargets shown in (**C**,**D**) were extracted from images (**A**,**B**) by the first author, according to relevant copyright permissions. Due to copyright reasons, images used in the actual experiment cannot be displayed. These alternate representative images were separately annotated by 5 independent subjects using an approach identical to Experiment 1.

Figure 1 illustrates the systematic expectations produced by humans on two example scenes: the first scene was rated by human subjects as likely to contain people but not cars, whereas the second was rated as likely to contain cars but not people. To measure the reliability of these expectations, we divided the subjects into two groups and calculated the correlation between the average rating obtained from each group across all images. All correlations were large and highly significant (r = 0.94, 0.9, 0.91, 0.89, 0.47 for likelihood, x-position, y-position, area and aspect ratio respectively between odd- and even-numbered subjects for cars; r = 0.87, 0.79, 0.96, 0.86 & 0.36 for people; p < 0.00005 for all correlations).

### Computational models for car and person likelihood

Next we asked whether the above systematic expectations can be predicted and understood using computational modelling. To this end we divided the image features present in each scene into target-related features, non-target objects and scene context features (see Methods). The inclusion of target-related features might appear counter-intuitive at first glance since these scenes do not contain target objects. However we included them nonetheless for completeness as well as because human expectations might still be driven by the weak presence of target-like features in a given scene. We tested a number of models based on combinations of target, nontarget and coarse scene information. Models were evaluated for their ability to predict the average likelihood ratings for novel scenes that were never used in model fitting (Table 1).

**Table 1:** Model performance on predicting car/person likelihood ratings in humans.

Model Name	Correlation with person likelihood	Correlation with car likelihood
*Ceil*	*0*.*87* ± *0*.*02*	*0*.*94* ± *0*.*01*
TNC	0.65 ± 0.01^#^	0.59 ± 0.01^#^
T	0.21 ± 0.02*	0.12 ± 0.02*
N	0.51 ± 0.02*	0.53 ± 0.01*
C	0.61 ± 0.01*	0.48 ± 0.01*
TN	0.54 ± 0.02*	0.52 ± 0.01*
TC	0.60 ± 0.01*	0.47 ± 0.01*
NC	0.65 ± 0.01	0.60 ± 0.01

*Ceil* refers to data reliability, which is an upper bound on model performance given the inter-subject variability in ratings (see text). The best model for predicting car and person likelihoods was based on nontarget and coarse scene features (NC). We calculated model performance as the average cross-validated correlation (*mean* ± *sd*) over 1000 random 80–20 splits of the scenes. Asterisks represent the statistical significance of the comparison with the NC model (*is p < 0.001, ^#^is p > 0.05). Statistical significance was calculated as the fraction of 1000 random 80–20 splits in which model correlation exceeded the best model. Note that model performance sometimes reduces after adding extra features because of overfitting. Abbreviations: T, N, C: Targets, Nontargets and Coarse features. TN = Targets & Nontargets, etc.

**Figure 2:**
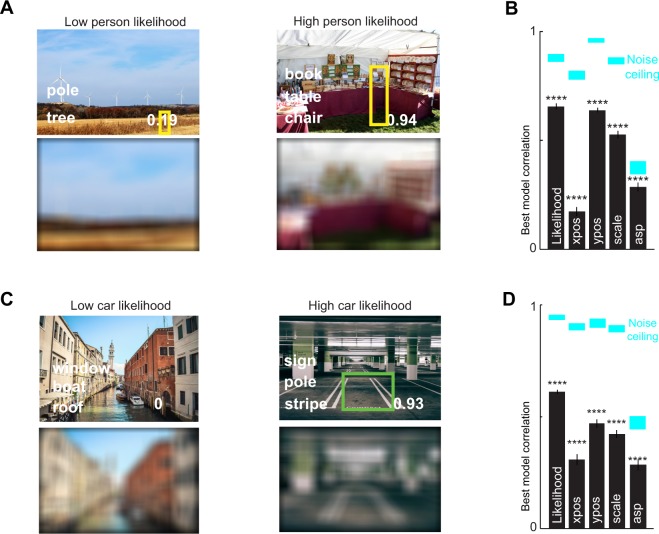
(**A**) Example scenes rated by subjects as having low and high person likelihood (top row) with nontarget labels and coarse scene structure (*bottom row*). Yellow boxes indicate the average location and scale at which a person was marked as most likely to occur in each scene by subjects (**B**) Correlation between best model (NC: nontargets and coarse scene features) predictions for likelihood, and the most likely horizontal position (*xpos*), vertical position (*ypos*), *scale* and aspect-ratio (*asp*) at which a person might occur in the scene. Cyan regions above each bar represent the reliability of the human data (mean ± std of corrected split-half correlation; see text). (**C**,**D**) Analogous plots for car likelihood data. Image sources (**A**) publicdomainpictures.net and yaketyyakyak@flickr (https://www.flickr.com/photos/yaketyyakyak/15312657660, CC BY 2.0) and (**C**) pixnio.com and pxhere.com. No changes were made for panels in top rows in (**A**,**C**) and coarse scene information was extracted by the first author and shown in bottom left and right panels in (**A**,**C**). Please click on the hyperlinks to open original images in a browser. These representative images were annotated by 5 independent subjects using the approach that was used to annotated 650 scenes drawn from Labelme^39^ and used for computational modeling in (**B**,**D**).

Overall, the best model for likelihood ratings was the one containing nontarget and coarse scene but not target features. We determined it to be the best model because (1) it yielded better fits to the data than models trained with only target, nontarget or coarse scene features (p < 0.001 in all cases). (2) It outperformed models based on other pairs of feature channels i.e. target and nontarget (p < 0.001 in both cases) or target and coarse scene structure (p < 0.01 in both cases) (3) its performance was equivalent to the full model containing target, nontarget and coarse scene features (p > 0.05). All values are given in Table 1. The performance of the best model is illustrated along with example scenes in Fig. 2. We also confirmed that these car and person likelihoods were predicted much better by coarse scene features compared to scene category labels alone (Table S5).

We then asked whether nontarget objects which increase car likelihood, also decreased person likelihood and vice-versa. For this analysis, we extracted regression weights for nontarget object labels in models that predicted person likelihoods and plotted them against regression weights for the same nontarget labels in models that predicted car likelihood. We obtained a negative and significant correlation confirming this prediction (r = −0.31, p < 0.05). We observed that nontargets such as *signage*, *cables* that frequently occur on highways tend to increase car likelihood and decrease person likelihood. Conversely, nontarget labels such as *bench*, *stair* and *cycle* tend to increase person likelihood and decrease car likelihood. Both patterns are as expected given the associations of these objects with cars and people respectively.

### Computational modelling of likely location, scale and aspect ratio

Next we asked if models based on combinations of target, nontargets and coarse scene features could predict other aspects of the likelihood data, namely the average horizontal location, vertical location, scale (i.e. area) and aspect ratio (i.e. vertical/horizontal extent) indicated during the likelihood task by human subjects. We visually inspected the annotated boxes that that subjects had drawn to indicate likely car or person locations and found that the average horizontal or vertical locations are meaningful in all but few exceptions such as when subjects draw boxes corresponding to likely person locations on either of two deck chairs and the average person box ends up being in the middle of two chairs. The results are summarized in Supplementary Table 1. In general models containing nontarget and coarse scene information (NC) yielded the best predictions (Fig. 2). Model predictions were significantly correlated with the observed human data but, fell short of the noise ceiling (Fig. 2), indicating differences in the underlying features used by humans and models.

Interestingly, models were better at predicting the vertical position of cars or people compared to horizontal location. This could be because vertical locations of cars/people vary less than horizontal locations, or because horizontal locations are harder to predict since its variations are due to differences in 3d scene layout. We note that the difficulty of predicting horizontal object locations has been reported previously^16^.

### Comparison with other computer vision models

**Figure 3:**
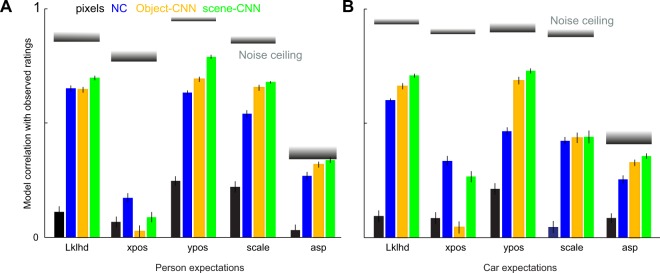
Comparison with other models. (**A**) Model performance on person likelihood data for raw pixels (black), nontarget + coarse scene features (blue), object-CNN (orange) and scene-CNN (green). The object-CNN was pre-trained for 1000-way object classification and the scene-CNN was pre-trained for 205-way scene classification. Shaded gray bars represent the noise ceiling for each type of data (mean ± std). (**B**) Model performance for car likelihood data. *Lklhd*: Likelihood.

To confirm the validity of our models and the specific choice of the feature channels, we compared the performance of the best model (NC) with the performance of three other models: (1) a pixel-based model in which image pixels are used directly as input; (2) a CNN pre-trained for 1000-way object classification^21^ and (3) a CNN pre-trained for scene classification^28^. The NC model yielded similar but slightly lower performance compared to the CNNs on predicting likelihoods, vertical position and scale but was better able to predict the expected horizontal location of targets (Fig. 3). All model predictions again fell short of the noise ceiling of the human data, indicating systematic differences in the underlying feature representations between models and humans.

### Augmenting deep networks with human-derived context expectations

The above results show that humans form highly systematic expectations about the overall likelihood, location and scale at which cars or people might occur in a scene, and that these expectations are largely driven by coarse scene features and the presence of nontarget objects. The fact that human expectations could be predicted using computational modelling meant that we could use these models to generate predicted human expectations without requiring any laborious manual annotations by human subjects.

In this section, we asked whether augmenting deep neural networks with these human-derived priors would improve their performance. An affirmative answer would indicate that contextual representations are qualitatively different in deep neural networks and humans. This method of combining decisions or scores learnt by separate models is called late-fusion^29,30^ and is appropriate in cases where beliefs or scores being fused are known to arise from different decision making processes.

We trained linear classifiers using feature vectors formed by concatenating confidence score from each CNN for the target category together with the predicted human expectations (likelihood, horizontal and vertical positions, scale and aspect ratio) generated for novel scenes without human annotations. To generate these predictions, we used the context-only model that was trained with coarse scene features alone as it explains most of the variance in the human ratings (Tables 1–2, Supplementary Tables 1 and 3). We also choose this approach as it can be scaled to large image databases where it is impractical to obtain human annotation of non-target objects. The resulting model performance is summarized in Table 2.

**Table 2:** Improvement in car/person detection obtained by augmenting state-of-the-art CNNs with predicted human-derived contextual expectations.

CNN	Target	CNN	CNN + Lklhd	CNN + yLocn	CNN + scale	CNN + Lklhd + yLocn + scale	CNN + all car & person ratings	Increase in %
RCNN^3^	*C*	82.4 ± 0.000	85 ± 0.000	82.5 ± 0.000	83.2 ± 0.000	85.5 ± 0.001	**86**.**2** ± **0**.**0017**	**3**.**8**
	*P*	80.6 ± 0.000	80.6 ± 0.001	81.5 ± 0.001	80.6 ± 0.000	80.4 ± 0.0018	**82**.**0** ± **0**.**0023**	**1**.**4**
Alexnet^2^	*C*	83.5 ± 0.000	85.8 ± 0.0012	83.5 ± 0.001	84.3 ± 0.0015	86.8 ± 0.0017	**87**.**1** ± **0**.**0019**	**3**.**6**
	*P*	73.4 ± 0.002	73 ± 0.0028	77.1 ± 0.0025	75.0 ± 0.0025	76.8 ± 0.0034	**77**.**1** ± **0**.**0038**	**3**.**7**

Each entry shows the cross-validated accuracy for detecting cars (C) or people (P) on novel scenes from the ADE20K dataset chosen from the same scene categories as in the human experiments (for details, see Supplementary Tables S2, S3 and S4). The best performing models are highlighted in bold. Columns indicate the kind of model used: the column marked CNN indicates the baseline accuracy of the deep neural network; the columns of the form “CNN + X” indicate accuracy for CNN augmented with feature X. Lklhd: predicted likelihood of target category object; xLocn: predicted horizontal location of target category object, yLocn: predicted vertical location of target category object; scale: overall bounding box area marked by subjects.

The augmented models perform uniformly better with better performance on scene categories shared with our original dataset (Table 2) and showed a modest improvement even on the full dataset (Table S3). As expected, the greatest improvement was obtained on the same set of scenes that were used in the human behavioural experiments (Table S4). The improved accuracy was not merely a result of adding more parameters since the accuracy is cross-validated (Table 2). Intuitively, accuracy benefits should arise only when the two-class separability is increased due to the additional dimension of predicted human priors. This can be seen in the case of RCNN posterior probability scores where specific attributes such as predicted car likelihood or person y-location increase classifier accuracy (Supplementary Fig. 1). This further indicates that accuracy benefits from augmentation do not arise due to overfitting.

**Figure 4:**
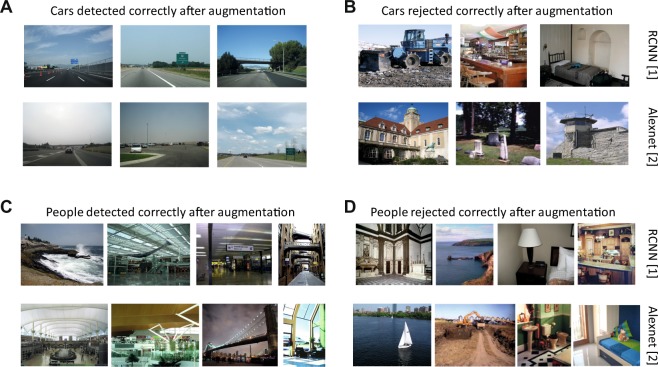
Augmenting CNNs with human expectations helps to accept low confidence detections (left) and reject false alarms (right). (**A**) Scenes containing small and hard to detect cars, these scenes are correctly classified as containing cars after augmentation with human derived priors (**B**) car false alarms that are correctly rejected after augmentation with human derived priors. (**C**) Scenes with multiple people at small scales and unusual locations (**D**) scenes devoid of people but falsely classified as person present by CNNs. All images selected from the ADE20K^4^ dataset and are best seen in high resolution in the digital version.

Example scenes that contain cars at scales and locations that make them hard to detect reliably are shown in Fig. 4. These scenes were classified correctly by augmenting CNN decisions with human derived priors. We find that scenes with box like objects can result in false alarms for cars, that are then effectively suppressed by incongruent scene layouts such as the abbey tower, building façade and bar counter scenes (Fig. 4B). Likewise, we find CNNs miss out people in many scenes (Fig. 4C) when people are present at very small scales or eccentric locations, such scenes also benefit from augmentation. Like in the case of cars, we find that incongruent contexts can also suppress false alarms like in the case of the river scene with a sailboat or outdoor farm scene with a tractor (Fig. 4), in both cases the presence of people at large scales is ruled out.

**Figure 5:**
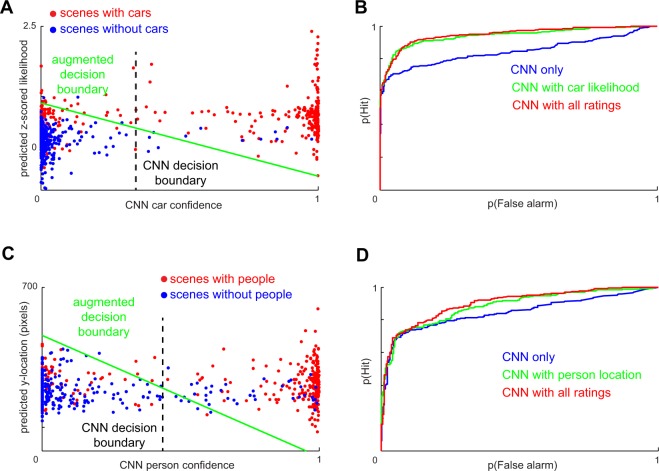
Augmenting CNNs with human expectations improves performance. (**A**) Classifier boundaries before (dashed line) and after (green) augmenting a CNN with predicted car likelihood ratings (**B**) ROC curves for the CNN, CNN with car likelihood & CNN with all car ratings. (**C**,**D**) Analogous plots for person detection augmented by predicted vertical location.

To further elucidate why CNN accuracy is benefited by augmenting with human contextual expectations, we plotted the predicted car likelihood for each scene against the baseline CNN confidence scores for the car category (Fig. 5A). The augmented classifier boundary has a negative slope that results in better performance. This performance improvement can be attributed to weak matches on high-likelihood scenes being correctly declared as targets, and strong matches on low-likelihood scenes being correctly rejected as a non-target. This improvement can be seen also in the ROC curves obtained by varying the decision criterion for the original CNN and the augmented CNN (Fig. 5B). We obtained similar improvements by augmenting baseline CNN person scores with predicted vertical location, and with all human-predicted priors combined (Fig. 5C,D; Table 2).

Could these performance benefits have been obtained simply from knowing the scene category? This is possible since the presence of categories often indicate the presence of certain diagnostic objects such as kitchens indicating ovens. To investigate this possibility, we trained separate models to perform car classification and person classification using manually annotated scene labels specified in ADE20K^4^. We then applied these models separately to novel scenes in ADE20K^4^ and used the scene-wise posterior probabilities to augment CNN decisions on these novel scenes. These manual scene category labels do improve CNNs but not as much as human derived priors (Table S6). We would like to emphasize here that scene label derived priors require a necessary manual step where human annotators must not only assign scene category labels on all the novel scenes but also have good consensus on the same. Our human derived priors require assignment of generic object related expectations to a small set of 650 reference images.

### Does augmenting improve accuracy on other categories as well?

Could augmenting CNNs with car/person expectations improve accuracy on other categories as well? Such an improvement is plausible for two reasons: first, many objects (e.g. bottle, train) are strongly associated with people and second, these objects may occur rarely even in large datasets, leading to poor classification rates. We tested this idea by augmenting CNN confidence scores for a number of additional categories using predicted car/person expectations as before. Remarkably, we obtained an improvement in classification accuracy of 3–20% on a number of categories from the Pascal VOC challenge set^31^ (Table 3), on scenes that closely matched our reference set of 650 car-person absent scenes (scene categories detailed in^5^). Since many of these classes are rare even in the relatively large ADE20K dataset^4^, our results show that augmenting with human priors can provide benefits beyond the categories for which human annotation was obtained and amortize the effort needed to obtain human priors for few categories.

**Table 3:** Improvement in accuracy for other object categories.

Category	#Scenes	Alexnet	Alexnet + Car & Person ratings	Improvement in %	RCNN	RCNN + Car & Person ratings	Improvement in % pts
Airplane	77	81.0	81.0	0.0	68.8	72.5	3.7
bicycle	261	64.5	77.5	13.0	54.4	73.4	19.7
Bird	40	52.3	71.8	19.4	58.8	65.7	6.9
Bottle	13	59.2	77.8	18.5	79.5	79.5	0.0
Bus	202	53. 7	67.8	14.2	75.1	77.7	2.8
Chair	14	85.7	89.4	3.7	68.8	68.8	0.0
Dog	53	58.8	68.3	9.5	43.6	63.8	20.2
Horse	22	68.8	70.5	1.7	45.8	51.7	5.9
Motor	20	62.4	78.9	16.5	70.8	79.4	8.6
Pot	139	54.5	74.2	19.7	84.5	89.3	4.8
Couch	402	83.8	85.9	2.1	83.0	85.4	2.4
Train	21	75.8	77.5	1.7	76.7	76.7	0.0
Tv	226	73.4	80.1	6.7	80.0	83.7	3.7

Here too, two types of CNN object detectors: Alexnet^2^ and RCNN^3^ were augmented using human-derived car/person likelihood scores on novel scenes.

Why do some categories benefit by augmenting with human-derived expectations but not others? We discovered two systematic patterns. First, categories with low baseline CNN performance might benefit more by augmentation. This was indeed the case as evidenced by a significant negative correlation between accuracy improvement and baseline CNN accuracy (r = −0.87, p = 0.00013 for Alexnet^2^ and r = −0.71, p = 0.007 for RCNN^3^ across the 13 PASCAL^31^ categories tested). Second, categories strongly associated with people or cars – such as bicycle – might benefit by augmenting with human-derived people/car expectations. To assess this possibility, we calculated for each object category the conditional probability of it occurring when a car was also present: p(object present|car present). If that object is associated with the presence of a car, its probability will be larger or smaller than the probability p(object present) across the dataset. We took the absolute difference between these two quantities therefore as a measure of association between each category with cars, and likewise calculated a similar association index for people as well. The average association index (across cars and people) was significantly correlated with the augmentation benefit (r = 0.68, p < 0.005 across 13 categories). Thus, objects that are strongly associated with cars and people experience a greater benefit by augmenting with human expectations for cars and people. Our findings agree with recent approaches in machine learning where model parameters for different classes in a dataset can share information^32^ and this can be exploited to leverage representations learnt on frequent classes and then improve recognition performance on rare ones.

## Discussion

Our main finding is that machines and humans learn qualitatively different contextual representations. Specifically, we have shown that (1) Humans form systematic expectations about the likelihood, scale and position of potential target objects in scenes entirely lacking the object of interest; (2) These expectations can be learned using computational modelling, and can be used to augment state-of-the-art CNNs to improve performance; (3) This improvement was due to relatively poor matches at highly likely locations being correctly labelled as target and conversely strong matches at unlikely locations being correctly rejected as false alarms; and (4) This benefit is non-trivial in that it cannot be obtained by simply augmenting popular CNNs with other types of human responses or other computational models (see below).

The fact that state-of-the-art object detectors can be improved by augmenting them with human likelihood ratings raises several interesting questions. First, is the improvement substantial? We have observed an improvement of 4% in accuracy on scenes from similar categories as those used to learn human priors (Table 2), and about 1% improvement across all scenes (Table S3), and much larger improvements for other human-associated categories (Table 3). Such improvements may appear modest, but it must be emphasized that the baseline CNNs are already state-of-the-art and subsequent efforts to improve them have obtained similar improvements. For instance, the performance difference between the best and second-best algorithms is only 1%, as obtained from

http://host.robots.ox.ac.uk:8080/leaderboard/displaylb.php?challengeid=11&compid=1 on 27 July 2018. Previous attempts at improving class-wise categorisation using contextual information^33^ have also obtained 2% improvements over 20 PASCAL VOC classes (Table 3 in that study). Similar improvements have been obtained with CNNs trained separately on foreground objects and background information^34^ (3.6% improvement using contextual information: FGNet + BGNet over original scenes alone OrigNet, Guided combination, Table 4 in that study).

Second, what about augmenting object detectors directly with human performance during object detection itself? Human priors have been studied previously using gaze locations recorded while people search for targets^19^. In these tasks, more fixations are observed when people take longer to find the target, and these fixations can be predicted using scene gist. This raises the possibility that learning from human behaviour (eye position/response times) during object detection could produce similar gains in performance as observed with the human likelihood ratings. To address this issue, we used data from a previous study in which we measured the response times of humans during target detection on the same scenes^5^. Interestingly, observed response times were uncorrelated with observed car likelihood ratings (r = 0.005, p = 0.9) and only weakly correlated for person likelihood ratings (r = 0.2, p < 0.005). Thus detection times are qualitatively different from likelihood ratings. It is important to note here that these response times had a clear category specific component^5^. To investigate this further, we trained models to predict detection response times, and generated their predictions on novel scenes from ADE20K^4^. Augmenting CNNs with these predictions barely improved performance (accuracy improvement: 0.34% for car, 0.87% for person), in contrast to the ~3% increase observed using likelihood predictions. We speculate that these gains are only incremental because detection times are strongly determined by target features^5^ and only weakly by priors, and that target features are already captured reasonably well by CNNs.

Third, can the same performance benefits be obtained by augmenting CNNs with other models trained on target features or even target present scenes? To investigate this issue, we augmented CNNs with predictions of HOG-based models trained for car/person classification using a standard set of target-present and target-absent scenes. This yielded only a slight improvement in top-1 performance (0.4% for car & 0.1% for person) compared to the ~3% increase observed with human-derived priors (see Methods for details).

Fourth, can similar performance benefits be obtained if CNNs are trained separately on target and background information? Recent studies suggest the answer to be in the affirmative. Specifically, training deep networks separately on object-occluded scene context and isolated objects and then combining their responses leads to better performance compared to models trained on full scenes^34^. To assess whether background information learned by object-occluded deep networks is similar to that learned by humans, we augmented both Alexnet^2^ and RCNN^3^ network decisions with class probabilities derived from models trained with only object-occluded scene features^34^. Models were trained for car/person classification using a standard set of car-present and person-present scenes (see Methods for details). This yielded only modest gains in performance (average improvement: 0.25% across car and person classes), again suggesting that human contextual expectations are different. Additionally, we found that class probabilities of these models were weakly correlated with human likelihood ratings in target-absent scenes (r = 0.24, p < 0.00005 for car; r = 0.21, p < 0.00005 for person). Thus, humans seem to have learned qualitatively different features compared to deep networks trained on object-occluded context information. We note that even auxiliary tasks such as person action recognition^35^, object segmentation^36^ and predicting missing or wrongly located objects^37^, benefit when background regions are sampled separately. These studies complement our observation that augmenting object CNNs with human-derived context models can improve performance. We also speculate that models representing object and contextual information separately may also be more immune to overfitting to target features as is known to happen with very deep convolutional networks^2^.

Finally, it could be argued that training machine vision algorithms with larger datasets may enable them to learn human-like priors as well. We consider this unlikely since even large-scale image^27^ and scene datasets^4^ contain the same kind of training data (positive and negative examples) that make it difficult to learn informative context signals. Moreover, deeper architectures may not necessarily help, because they are biased towards target features rather than coarse scene layout^2^. Increase receptive field sizes arising from successive layers of pooling might also cause context and target information to get more entangled. However, the finding that training separately on background and foreground can improve overall performance^34^ is concordant with our results. The existence of separate brain regions for processing object and scene information also supports the argument that foreground and background must be treated independently.

We surmise that there are more effective ways of integrating such human priors into deep convolutional architectures. Some promising avenues are separating objects from context^34^, attentional modules^3^ and incorporating scale priors using skip layers^38^. It is possible that attentional mechanisms in humans are also optimized to yield benefits in object detection, since this is a core function of the human visual system.

## Methods

### Participants

Eleven subjects (3 female, 20–30 years old) participated in the task. All subjects had normal or corrected-to-normal vision and gave written informed consent to an experimental protocol approved by the Institutional Human Ethics Committee of the Indian Institute of Science, Bangalore. All methods were performed in accordance with the relevant intuitional guidelines and regulations.

### Stimuli

For human behavioural experiments, we selected a total of 650 full colour real-world scenes with a resolution of 640 × 480 pixels (spanning 13.5° by 10.1° visual angle) containing neither cars nor people and have been used in an earlier study^5^. A large fraction of these scenes were from the LabelMe^39^ and were used in a previous fMRI study^40^, and the rest were from a personal collection of one of the authors (M.V.P). Scenes included a wide range of natural and urban environments spanning many common scene categories (airport terminal, beach, botanical garden, bridge, coast, forest road, orchard, bamboo forest, bus station, cottage garden, driveway, forest, forest path, highway, hill, mountain, mountain path, mountain road, park, parking lot, picnic area, playground, rainforest, residential neighbourhood, river, runway, shipyard, ski lodge, ski resort, stage, taxiway, train station, tundra, valley, vegetable garden, village, waterfall, wheat field, woodland, workroom, parade ground). These scene categories are also well represented in the ADE20K dataset^4^ which we have used for subsequent computational experiments. These 650 scenes also contained a variety of non-target objects. The number of times these objects occurred in these 650 scenes were: window (332), tree (327), pole (267), door (160), fence (149), sign (147), roof (147), text (103), lamppost (90), glass (82), cable (80), stripe (58), box (56), bush (47), stair (45), bench (42), rock (41), dustbin (36), flower-pot (35), lamp (29), flower (26), chair (26), entrance (23), cycle (22), table (20), boat (19), statue (17), hydrant (8), flag (8), wheel (7), animal (7), cone (6), bird (6), manhole-cover (5), cloud (5), bag (2).

### Procedure

Subjects used a custom GUI interface created in Matlab®. They were instructed to assess how likely they thought a target could occur in the real scene if it was observed for a long time. They had to indicate this using a slider bar on the screen (with the two ends marked “very likely” to “very unlikely”). For every scene rated with non-zero likelihood for a category, the subject was asked to place a rectangular box to mark the most likely location and size at which the target would occur in the scene. For each scene, subjects had to indicate this for two target categories: cars and people in any order. The likelihood ratings were converted into a probability score by scaling them into the interval [0 1].

### Computational modelling of human expectations

To understand the features that underlie human expectations, we extracted distinct types of visual information from each scene: targets, nontargets and scene context. Our approach is described and validated in detail elsewhere^5^ and is summarized briefly below.

### Target features

We extracted a total of 61 features from each scene. These features are templates of the visual appearance of cars and people across typical views and have been learned using an independent set of close cropped car and person images. We employed six models (2 categories x 3 views) based on Histograms of Oriented Gradients (HOG), which have been used previously to detect cars and people^33^. On convolution of the learned template with a scale pyramid of the scene, strong matches result in hits. We first thresholded the degree of match between the car/person template and a scene region at two levels, one is a tight threshold of −0.7 that has very few false alarms across the entire dataset and a second weaker threshold of −1.2 is set to allow for correct detections as well as false alarms. A diverse set of 31 attributes was extracted separately, once for car and once for person. These included the number of hits (n = 1 feature) at high detector confidence s(>−0.7), estimate of false-alarms (n = 1 feature) computed as the difference between number of detections at strong (>0.7), average scale (area) of detected box (n = 1 feature), and weak partial matches (>−1.2). Part-deformation statistics (n = 16 features) were calculated by first normalising each detection to a unit square and finding the displacement of each detected part from the mean location of the part across all scenes in ours dataset. We also included eccentricity (n = 5 levels from center of scene) and frequency of detected model types (n = 6, 2 categories x 3 views). Finally, an average detection score (n = 1) was extracted from HOG detections in a scene. Feature vectors for car and person were then concatenated and used as the target feature vector (n = 62). We found this summary of target features to be more informative than HOG histograms^22^ computed on the same detected locations.

### Nontarget features

These comprised binary labels corresponding to the presence/absence of the full set of objects that occurred across the set of 650 scenes. We avoided extracting image features from these objects since these could potentially be shared with target features. We explored the possibility of testing automated object detection using deep neural networks^21,41^, but this yielded too many erroneous labels that would compromise model predictions. Example nontarget labels are shown in Figs 1–2. Some representative nontarget labels and their frequency in the dataset is, window (332), tree (327), pole (267), door (160), fence (149), sign (147), roof (147), text (103), lamppost (90), glass (82), cable (80), stripe (58), box (56), bush (47), stair (45), bench (42), rock (41), dustbin (36), flower-pot (35), lamp (29), flower (26), chair (26), entrance (23), cycle (22), table (20), boat (19), statue (17), hydrant (8), flag (8), wheel (7), animal (7), cone (6), bird (6), manhole-cover (5), cloud (5), bag (2).

### Coarse scene features

These consisted of a combination of features encoded by the fc7 layer of a state-of-art deep convolutional network (CNN) optimized for scene categorisation^28^ together with the coarse spatial envelope GIST operator^18^. We included GIST features because they improved model predictions for horizontal locations of objects and marginally improved overall performance. In both cases, features were extracted by giving as input to each model a blurred version of the scene. The blurred scene was obtained by convolving the original scene with a low pass Gaussian filter (σ = 20 pixels), such that objects and their parts were no longer recognizable. To confirm that target or nontarget information was no longer present in these images, we took blurred scenes with and without cars/people and asked whether object-based detectors^33^ could correctly identify the scenes containing targets. This yielded poor detection accuracy (average accuracy: < 5% for both car and person detectors across 100 randomly chosen scenes).

### Model fitting and performance evaluation

We sought to assess whether human likelihood judgments on scenes could be predicted using target, nontarget and coarse scene features or a combination of these channels. To this end we fit models based on every possible subset of these channels. To identify the best model, we selected the model that outperformed all other models in terms of the match between observed likelihood ratings and cross-validated model predictions. We equated the complexity of each feature channel by projecting each subset of features along their first 20 principal components. This typically captured over 85% of variance across 650 scenes for each of the three information channels and provided a compact description of the features in each channel.

All models were fit with linear regression of the form y = Xb, where y is the vector of likelihood ratings (likelihood/x-location/y-location/scale/aspect-ratio), X is a matrix whose rows contain features for each scene derived from targets, nontargets and coarse scene structure and b is a vector of unknown weights representing the contribution of each column in X. We used standard linear regression to solve this equation. We tested all models for their ability to predict average ratings on novel scenes using 5-fold cross-validation. All models were trained and tested on scenes that were devoid of cars as well as people and hence only predict the human beliefs about car or person attributes such as likelihood of presence, location or scale. We concatenated model predictions on the cross-validation test sets and calculated the correlation with the observed ratings obtained from the behavioural experiment. A perfect agreement between predicted and observed ratings would yield a correlation coefficient of 1 with a high statistical significance (i.e. p < 0.05 of observing this correlation by chance). In contrast, non-informative model predictions would result in near-zero correlations that are typically not statistically significant.

### Noise ceiling estimates

To estimate an upper bound for model performance, we reasoned that model performance cannot exceed the reliability of the data. We estimated this reliability by calculating the correlation coefficient between average per-scene ratings between two randomly chosen groups of subjects, and applying a correction to account for the fact that this correlation is obtained between two halves of the data rather than on the full dataset. This correction, known as the Spearman-Brown correction, is given by *rc* = *2r/(r* + *1)*, where *r* is the split-half correlation.

### Augmenting CNNs with human-derived expectations

We selected two state-of-the-art CNNs for testing. The first CNN was similar to the BVLC reference classifier^2^ that has a mean average precision (mAP) of 72% on the PASCAL VOC 2007 dataset^31^. Hereafter we refer to this CNN as Alexnet. The second CNN has an inbuilt attention module and generates region proposals on which detection is carried out^3^: this model has 73.2% mAP on the same dataset^31^. We gave the highest possible benefit to this model by selecting the most confidently detected instance within every scene and for each category. Hereafter we refer to this CNN as RCNN. We used the RCNN implementation provided by its authors and this network included a VGG-16^42^ based classification module.

To evaluate object detection performance, we used images from the recently released ADE20K scene dataset^4^. This dataset contains over 20,000 real-world scenes with 5601 scenes containing people and 3245 scenes containing cars. The chosen scenes have high variability in composition of scenes as well as visual attributes of targets. For negative examples, we randomly sampled matching sets of car absent (n = 3245) and person absent scenes (n = 5601). We also selected a restricted subset of 372 scenes from the 3470 scenes containing cars, by visually matching scene types present in our reference set of 650 car-person absent scenes (see Methods). Likewise, we also selected a subset of 306 scenes from the larger set of 5601 scenes containing people. We also verified that the frequency with which human annotated scene category labels occurred in ADE20K^4^ (995 unique categories for car scenes, 1437 unique categories for person scenes), closely followed that observed in 650 scenes we used to obtain human ratings (r = 0.96, p = 0 for cars and r = 0.95, p = 0 for people between object label frequency in 650 scenes and that in the car present and person present scene sets we sampled from ADE20K^4^). We have further summarized these selection choices in the Supplementary Table 2.

### Augmenting CNNs with other model priors

We evaluated the benefits of augmenting Alexnet^2^ and RCNN^3^ decisions with posterior probabilities of models trained with either HOG based target appearance^5^ or object-cropped background information alone^34^. We trained models for car/person classification using a standard set of 1300 scenes, half of which contained the target object. We have used these scenes in a previous study^5^ and the 650 car-person absent scenes have been annotated by human participants in this study. Feature vectors (62 dimensional for HOG based features^5^, 4096 fc7 features for object-cropped BG features^34^), were extracted for each scene and models were trained for binary classification using 5-fold cross-validated linear discriminant analysis using Matlab©, classify and accompanying custom scripts. Car and person class probabilities were then obtained from these models for evaluation scenes from the ADE20K^4^ dataset.

## Supplementary information

Supplementary text
