# Supplementary material for: Machine vision benefits from human contextual expectations
Source: Sci Rep. 2019 Feb 14;9:2112. doi: 10.1038/s41598-018-38427-0 (PMC6375915; doi:10.1038/s41598-018-38427-0)
Supplement: Supplementary file 1 — Supplementary text [file 41598_2018_38427_MOESM1_ESM.docx]

**Supplementary information for**

**“Machine vision benefits from human contextual expectations”**

by

Harish Katti*, Marius V. Peelen, and S. P. Arun

**Supplementary Figures**


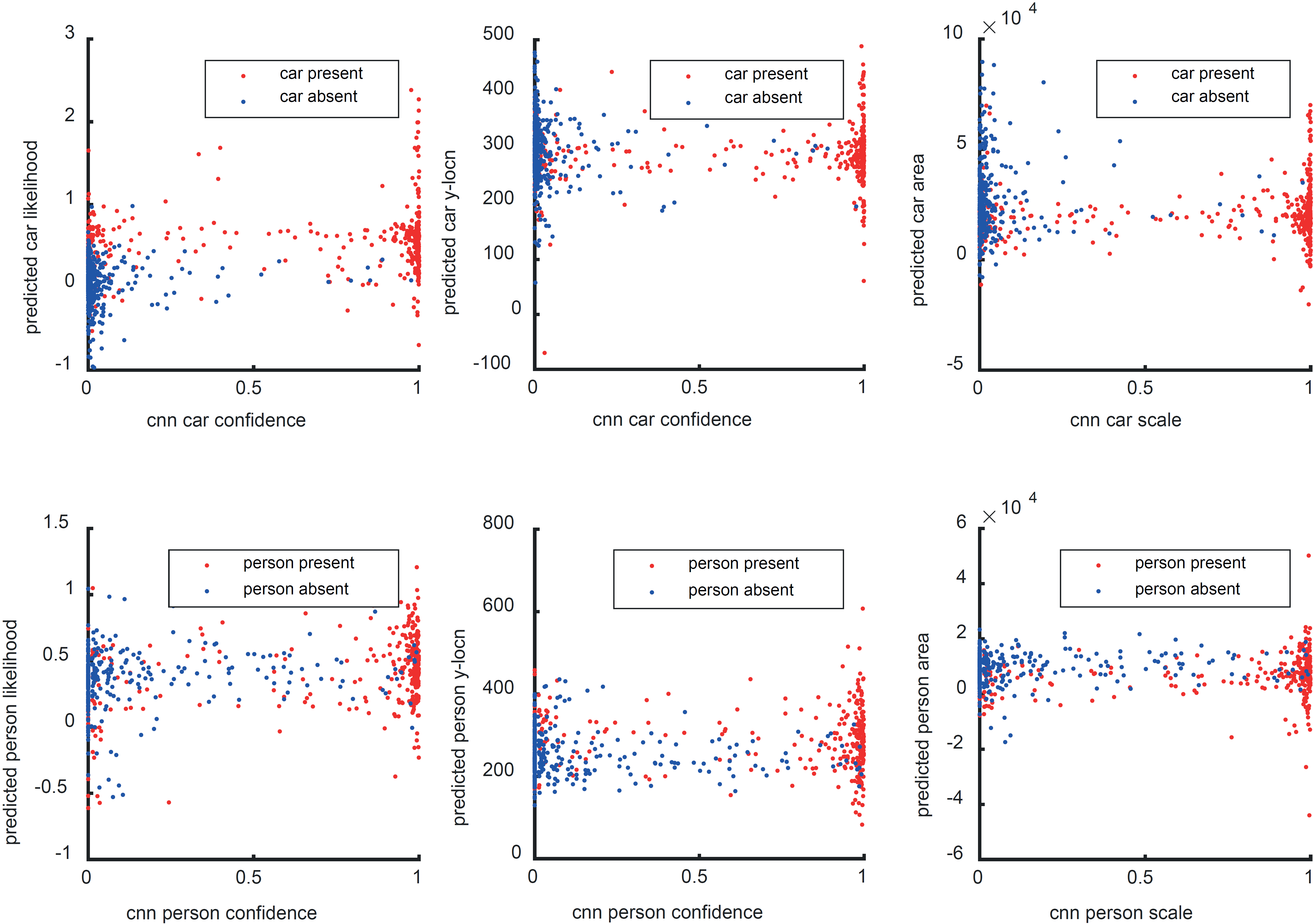


**Figure S1: Augmentation with some predicted human derived priors such as predicted car likelihood (top leftmost panel) or person y-location (bottom middle panel), bring about additional separability between target present and target absent scenes. This can be seen as the separation between red and blue dots along the Y-axis. This benefit is not merely a result of adding extra parameters as this extra separability is not seen for predicted human derived priors such as predicted car area (top rightmost) or person likelihood (bottom leftmost). This indicates that accuracy benefits from augmentation do not arise due to overfitting.**

**Supplementary Tables**

| **Model** | **Person data** | | | | **Car data** | | | |
| --- | --- | --- | --- | --- | --- | --- | --- | --- |
|  | **xpos** | **ypos** | **scale** | **asp** | **xpos** | **ypos** | **scale** | **asp** |
| *Ceil* | *0.79±0.02* | *0.96±0.01* | *0.86±0.01* | *0.36±0.03* | *0.9±0.01* | *0.91±0.2* | *0.89±0.02* | *0.47±0.03* |
| **TNC** | 0.17±0.02# | 0.63±0.01# | 0.52±0.02* | 0.28±0.02# | 0.30±0.02 | 0.45±0.02# | 0.41±0.02# | 0.27±0.02# |
| **T** | 0.00±0.02* | 0.10±0.02* | 0.09±0.02* | 0.09±0.02* | 0.10±0.03* | 0.08±0.02* | 0.08±0.03* | 0.14±0.02* |
| **N** | 0.10±0.02* | 0.40±0.01* | 0.44±0.01* | **0.29±0.01** | 0.03±0.03* | 0.21±0.02* | 0.22±0.02* | 0.13±0.02* |
| **C** | **0.18±0.02** | 0.58±0.01* | 0.46±0.01* | 0.19±0.02* | **0.33±0.02** | 0.43±0.01 | **0.42±0.02** | **0.30±0.02** |
| **TN** | 0.09±0.02* | 0.40±0.01* | 0.42±0.02* | 0.27±0.02# | 0.00±0.02* | 0.24±0.02* | 0.24±0.02* | 0.17±0.02* |
| **TC** | 0.17±0.02# | 0.59±0.01* | 0.46±0.01* | 0.20±0.02* | 0.28±0.02* | 0.42±0.02 | 0.40±0.02# | 0.28±0.02# |
| **NC** | 0.17±0.02# | **0.63±0.01** | **0.54±0.02** | 0.29±0.02# | 0.33±0.02# | **0.46±0.02** | 0.42±0.02# | 0.30±0.02# |

**Table S1. Model performance on predicting likely location, scale and aspect ratio.** Models based on various combinations of features were trained separately to predict the horizontal position (xpos), vertical position (ypos), area (scale) and aspect ratio (asp) at which a person was most likely to occur in the scene. The best model in each case is indicated using **bold face**. All other conventions are as in Table 1.

| **Scene subsets from ADE20K**^1^ | **Attributes** | | | |
| --- | --- | --- | --- | --- |
|  | **#Scenes** | **#Car present** | **#People present** | **Scene categories** |
| **Car (C)** | 6940 | 3470 | 0 | All scene categories in ADE20K [3] |
| **Scene matched (C)** | 744 | 372 | 0 | Matched to scene categories for 650 scenes in behavioral experiment |
| **Person (P)** | 11202 | 0 | 5601 | All scene categories in ADE20K [3] |
| **Scene matched (P)** | 612 | 0 | 306 | Matched to scene categories for 650 scenes in behavioral experiment |

**Table S2. Scenes used to evaluate CNN augmentation with human derived priors.** The ADE20K^1^ dataset contained many scene categories that were qualitatively different from the scenes included in the human experiments. For instance, it is unreasonable to expect that human expectations learned from outdoor scenes would be relevant to indoor scenes. We therefore evaluated the performance of CNNs augmented with human priors on scenes from the dataset that belonged to categories that were similar to those used during the human experiments. The scene-matched categories included airport-terminal, alley, bridge, coast, driveway, forest-road, highway, mountain, park, parking-lot, playground, residential-neighborhood, resort, runway, ski-lodge, ski-resort, taxiway, valley, vegetable-garden and yard scenes. The performance in Table 2 of the main text is reported using these scenes (corresponding to the matched entries). We excluded outdoor scenes such as abbey, airport, aqueduct, artificial, auto-racing-paddock, barn, bathhouse-outdoor, booth-outdoor, cargo-deck, desert-road, drainage-ditch, earth-fissure, exhibition-hall, flood, guardhouse, hatchery, hospital, manufactured-home, military-hut, office-building, pantry, ramp, road, roof-garden, saloon, science-museum. Likewise we excluded indoor scenes such as bus-interior, living-room etc. The performance of the CNNs augmented with human priors on the entire dataset is nonetheless reported below in Supplementary Table S3.

| **CNN** | **Target** | **CNN** | **CNN + Lklhd** | **CNN +**  **yLocn** | **CNN +**  **scale** | **CNN+**  **Lklhd + yLocn +**  **scale** | **CNN +**  **all car & person ratings** | **Increase in %** |
| --- | --- | --- | --- | --- | --- | --- | --- | --- |
| RCNN^2^ | *C* | 88.3 | 88.3 | 88.3 | 88.3 | 88.4 | **88.6** | **0.3** |
|  | *P* | 84.0 | 84.6 | 84.0 | 84.0 | 84.8 | **84.9** | **0.9** |
| Alexnet^3^ | *C* | 82.5 | 82.6 | 82.4 | 83.0 | 83.3 | **84.0** | **1.5** |
|  | *P* | 76.8 | 77.8 | 76.7 | 76.8 | 78.2 | **78.7** | **1.9** |

**Table S3: Improvement in car/person detection obtained by augmenting state-of-the-art CNNs with predicted human-derived contextual expectations on all scenes from ADE20K**^1^**.** Each entry shows the cross-validated accuracy for detecting cars on all (C) or matched (CM) scenes from ADE20K, or people on all scenes from ADE20K – for details see Supplementary Table 1. Best models are highlighted in bold. Columns indicate the kind of model used: the column marked CNN indicates the baseline accuracy of the deep neural network; the columns of the form “CNN + X” indicate accuracy for CNN augmented with feature X. Lklhd: predicted likelihood of target category object; xLocn: predicted horizontal location of target category object, yLocn: predicted vertical location of target category object; scale: overall bounding box area marked by subjects.

| **CNN** | **Target** | **CNN** | **CNN + Lklhd** | **CNN +**  **yLocn** | **CNN +**  **scale** | **CNN+**  **Lklhd + yLocn +**  **scale** | **CNN +**  **all car & person ratings** | **Increase in %** |
| --- | --- | --- | --- | --- | --- | --- | --- | --- |
| RCNN | *C* | 87.54 | 87.22 | 87.54 | 87.33 | 87.47 | **90.36** | **2.82** |
|  | *P* | 84.30 | 85.54 | 85.98 | 86.09 | 85.55 | **88.23** | **3.93** |
| Alexnet | *C* | 80.49 | 80.65 | 80.98 | 80.61 | 81.28 | **86.23** | **5.74** |
|  | *P* | 64.19 | 68.86 | 69.77 | 74.08 | 72.62 | **79.91** | **15.72** |

**Table S4: Improvement in car/person detection with augmenting CNNs with predicted human priors on scenes partially used during training.** A total of 650 car-present scenes were added to the 650 scenes used in the human behavioural experiments to obtain a balanced set for car classification. Likewise, 650 person-present scenes were added to the same 650 scenes used for the human experiments to obtain a balanced set for person classification. These scenes are identical to those used in an earlier study^4^. To evaluate performance, we trained models to predict human car priors using 80% of the data and augmented RCNN and Alexnet scores on the held out 20% and repeated this process to cover the entire set of 1300 scenes. We followed a similar procedure for person classification as well.

| **Model Name** | **Correlation with person likelihood** | **Correlation with car likelihood** |
| --- | --- | --- |
| *Ceil* | *0.87±0.02* | *0.94±0.01* |
| TNCS | 0.64+/-0.01 | 0.61+/-0.01 |
| T | 0.21+/-0.01 | 0.12+/-0.02 |
| N | 0.52+/-0.02 | 0.53+/-0.01 |
| **C** | **0.59+/-0.01** | **0.48+/-0.00** |
| **S** | **0.12+/-0.01** | **0.18+/-0.01** |
| TNC | 0.65+/-0.01 | 0.60+/-0.01 |
| NCS | 0.64+/-0.01 | 0.62+/-0.00 |
| TCS | 0.58+/-0.01 | 0.50+/-0.01 |
| CNS | 0.64+/-0.01 | 0.62+/-0.00 |
| TN | 0.54+/-0.01 | 0.53+/-0.01 |
| TC | 0.58+/-0.01 | 0.47+/-0.01 |
| TS | 0.23+/-0.01 | 0.19+/-0.02 |
| NC | 0.65+/-0.01 | 0.61+/-0.01 |
| NS | 0.52+/-0.01 | 0.54+/-0.01 |
| **CS** | **0.58+/-0.01** | **0.52+/-0.00** |

**Table S5. Human expectations are poorly predicted by scene category labels.** To investigate how well human expectations can be predicted by scene category labels, we labelled the 650 scenes used in behavioural experiments with the same scene labels as in the ADE20K^1^ dataset and defined these human annotated scene labels as an independent channel of information (denoted by **S**). We then trained models with all combinations of target (T), nontarget (N), coarse scene information (C) and human annotated scene labels (S). Model performance was evaluated using 5-fold cross-validation. Models trained with coarse scene information perform just as well as those trained with a combination of coarse scene information and human annotated scene labels (C vs CS). This indicates that our coarse scene representations already contain the information about scene labels and are also more informative about human priors.

| **CNN** | **Target** | **CNN** | **CNN +**  **Human priors** | **Increase in %** | **CNN +**  **Scene priors** | **Increase in %** |
| --- | --- | --- | --- | --- | --- | --- |
| RCNN | *C* | 88.3 | 88.6 | **0.3** | 88.35 | **0.05** |
|  | *P* | 84.0 | 84.9 | **0.9** | 84.26 | **0.26** |
| Alexnet | *C* | 82.5 | 84.0 | **1.5** | 83.6 | **1.1** |
|  | *P* | 76.8 | 78.7 | **1.9** | 78.3 | **1.5** |

**Table S6: Augmenting CNNs with predicted human priors leads to greater improvements than augmenting CNNs with scene category labels.** Each entry shows the cross-validated accuracy for detecting cars on all scenes from ADE20K, or people on all scenes from ADE20K. Best models are highlighted in bold. Columns indicate the kind of model used: the column marked CNN indicates the baseline accuracy of the deep neural network; the columns of the form “CNN + X” indicate accuracy for CNN augmented with feature X. **Human priors**: combining with classifiers trained on predicted car and person likelihood of target category object, horizontal and vertical location of target category object, overall bounding box area marked by subjects. These are essentially the same as the final column in Table S3. **Scene priors:** Combining with the output of two classifiers trained on manually annotated scene labels, one for car classification on the independent set of 650 car present and 650 car absent scenes, another for person classification on the independent set of 650 person present and 650 person absent scenes. These models were then applied to novel scenes in the ADE20K^1^ dataset and posterior probabilities obtained were used as scene label derived priors to augment CNN decisions.

**REFERENCES**

1. Zhou, B. *et al.* Semantic Understanding of Scenes through the ADE20K Dataset. *arXiv* (2016).

2. Ren, S., He, K., Girshick, R. & Sun, J. Faster R-CNN: Towards Real-Time Object Detection with Region Proposal Networks. *IEEE Trans. Pattern Anal. Mach. Intell.* **PP,** 1–1 (2016).

3. Lapuschkin, S., Binder, A., Montavon, G., Müller, K.-R. & Samek, W. Analyzing Classifiers: Fisher Vectors and Deep Neural Networks. *2016 IEEE Conf. Comput. Vis. Pattern Recognit.* 17 (2016). doi:10.1109/CVPR.2016.318

4. Katti, H., Peelen, M. V. & Arun, S. P. How do targets, nontargets, and scene context influence real-world object detection? *Attention, Perception, Psychophys.* (2017). doi:10.3758/s13414-017-1359-9
